# Association of Blue Light–Filtering Intraocular Lenses With All-Cause and Traffic Accident–Related Injuries Among Patients Undergoing Bilateral Cataract Surgery in Finland

**DOI:** 10.1001/jamanetworkopen.2022.27232

**Published:** 2022-08-17

**Authors:** Piotr Kanclerz, Idan Hecht, Mariana Cunha, Boris Knyazer, Ilkka Laine, Raimo Tuuminen

**Affiliations:** 1 Helsinki Retina Research Group, University of Helsinki, Helsinki, Finland; 2 Hygeia Clinic, Gdańsk, Poland; 3 Sackler School of Medicine, Tel Aviv University, Tel Aviv, Israel; 4 Department of Ophthalmology, Shamir Medical Center, Tel Aviv, Israel; 5 Medical School, São Paulo State University, São Paulo, Brazil; 6 Department of Ophthalmology, Soroka University Medical Center, Beersheba, Israel; 7 Faculty of Health Sciences, Ben-Gurion University of the Negev, Beersheba, Israel; 8 School of Engineering, Aalto University, Helsinki, Finland; 9 Department of Ophthalmology, Kymenlaakso Central Hospital, Kotka, Finland

## Abstract

**Importance:**

Blue light–filtering (BLF) intraocular lenses (IOLs) have been widely used in clinical practice for more than 20 years and have been implanted in millions of patients with cataracts worldwide. However, little evidence on the association of BLF IOLs with injuries is available.

**Objective:**

To assess the association of BLF IOLs with all-cause and traffic accident–related injuries and quality of vision while driving after bilateral cataract surgery.

**Design, Setting, and Participants:**

This retrospective registry-based cohort study included patients who underwent bilateral cataract surgery between September 3, 2007, and December 14, 2018, and were followed until December 14, 2021. Surgery was performed at the Department of Ophthalmology, Kymenlaakso Central Hospital, Kotka, Finland. The 4986 participants received non-BLF IOLs (n = 2609) or BLF IOLs (n = 2377) in both eyes. Patients undergoing bilateral surgery between 2015 to 2016 with non-BLF IOLs (n = 102) or BLF IOLs (n = 91) and currently driving a car were interviewed using a structured questionnaire for visual performance while driving.

**Exposures:**

Follow-up for a mean (SD) of 2166 (1110) days after second eye surgery.

**Main Outcomes and Measures:**

Kaplan-Meier and multivariable Cox proportional hazards regression analyses for the risk of all-cause and traffic accident–related injuries after surgery in the second eye obtained from the patient medical records were assessed. To improve follow-up precision, both death and the end of the follow-up were used as censoring events.

**Results:**

A total of 4986 patients were included in the analysis (1707 [34.2%] men and 3279 [65.8%] women; mean [SD] age, 73.2 [8.6] years at the first surgery and 74.3 [8.8] years at the second). Injury-free survival rates preceding the first eye surgery were comparable between the non-BLF and BLF IOL groups (hazard ratio adjusted for age and sex, 0.95 [95% CI, 0.81-1.13; *P* = .57]). In multivariable Cox proportional hazards regression analysis controlling for age and sex, the use of BLF IOLs showed no advantage in overall injuries compared with the use of non-BLF IOLs (hazard ratio, 0.99 [95% CI, 0.88-1.11]; *P* = .85) or in any injury subtype. Subjective visual performance parameters for driving were all comparable between the non-BLF and BLF IOL groups except for glare when driving in the dark (evening or night), which occurred among 9 of 80 patients with BLF IOLs compared with 0 of 83 non-BLF IOLs (*P* < .001).

**Conclusions and Relevance:**

The findings of this cohort study suggest that use of BLF IOLs was not associated with reduced risk of injuries, whereas glare during nighttime driving was significantly worse in the BLF IOL group with pseudophakia.

## Key Points

**Question:**

Are blue light–filtering (BLF) intraocular lenses (IOLs) implanted during bilateral cataract surgery associated with improved outcomes regarding traffic accident–related injuries and driving safety?

**Findings:**

In this cohort study of 4986 patients who underwent bilateral cataract surgery within the follow-up period of more than 2000 days, neither cumulative all-cause nor traffic accident–related, injury-free survival differed between patients with BLF and non-BLF IOLs. Patients with BLF IOLs experienced more glare during nighttime driving, whereas other subjective disturbances were comparable between patients with BLF and non-BLF IOLs.

**Meaning:**

These findings suggest that no advantage was associated with BLF IOLs in terms of risk of injury and comfort with driving.

## Teaser

This cohort study analyzes the risk of injuries and visual quality for driving among patients who underwent bilateral surgery for cataracts and received implants with blue light–filtering or non–blue light–filtering intraocular lenses.

## Introduction

Laboratory and animal models have demonstrated that short-wavelength light (400-460 nm) causes retinal damage.^1,2,3^ In a healthy human lens, the total transmission of visible light decreases with age, and this decrease is more prominent for short wavelengths.^4^ Although most intraocular lenses (IOLs) absorb UV light, only blue light–filtering (BLF) IOLs have a chromophore that absorbs short-wavelength light. Blue light–filtering IOLs were designed to approximate light filtration of the natural, healthy crystalline lens and to protect the retina from high-energy wavelengths of the blue light spectrum.^5^

Blue light is only partially responsible for photopic vision but is critical for scotopic vision.^6,7^ The decrease in scotopic visual function, which is associated with aging, contributes to the increased risk of falling^8^ and difficulties in driving and reading road signs at night^9^ in elderly individuals. Single studies^10,11^ have reported that BLF IOLs provide similar or better postoperative contrast sensitivity at select frequencies when compared with non-BLF IOLs. On the other hand, there is significant concern that BLF IOLs might impair the ability to see in the dark.^12^ Furthermore, stray light, the cause of disability glare, was increased in patients with pseudophakia who received BLF IOLs compared with non-BLF IOLs.^13,14^ The aim of this study was to analyze the risk of injuries and visual quality for driving in patients with cataracts undergoing bilateral implants with non-BLF or BLF IOLs in both eyes.

## Methods

### Study Design

This retrospective, registry-based cohort study was approved by the Research Director and the Chief Medical Officer of the Kymenlaakso Central Hospital, Kotka, Finland, and adhered to the tenets of the Declaration of Helsinki. The study included consecutive cataract operations performed between September 3, 2007, and December 14, 2018, at the Department of Ophthalmology of the Kymenlaakso Central Hospital. All adult patients (aged ≥18 years) who underwent uneventful phacoemulsification surgery and in-the-bag implantation of non-BLF IOLs (ZA9003 and ZCB00/PCB00 [Tecnis]; Abbott Medical Optics Johnson & Johnson Vision, Inc) or BLF IOLs (SN60WF/AU00T0 [Acrysof]; Alcon Laboratories Inc) in both eyes were included (eFigure 1 in the Supplement). The Department of Ophthalmology at Kymenlaakso Central Hospital is a governmental, tax-financed unit covering the hospital district of approximately 175 000 inhabitants; patients’ medical records can be used for registry-based purposes. This report followed the Strengthening the Reporting of Observational Studies in Epidemiology (STROBE) reporting guideline.

All patients referred for cataract surgery are allocated to the treating physicians from the cataract surgery operation queue solely by the Kymenlaakso Central Hospital. The IOL type was assigned at the discretion of the surgeon and was not randomly allocated. The patient had no choice in the type of lenses implanted in their eyes. There were no financial arguments in selecting between the non-BLF and BLF IOLs. The clinical practice of the unit was to use the same IOL type for the contralateral eye. Both IOL types (non-BLF and BLF) were used throughout the study period. Both IOL types are made of acrylic and are of similar design, making them proper candidates to assess the associations of the BLF aspect, which is the key difference between them. Before surgery, all patients underwent a complete ophthalmological examination including best-corrected visual acuity evaluation, tonometry, slitlamp examination (which included anterior segment and fundoscopy assessment), and biometry.

The main outcome measure was the association of BLF IOLs with the incidence of all-cause and traffic accident–related injuries after the cataract surgery in the second eye. Exploratory analysis according to sex and age was performed as a post hoc analysis. A secondary outcome was the association of BLF IOLs with visual quality for driving using a structured questionnaire by an ophthalmic nurse, who was masked regarding the implanted IOL types.

### Injuries

Coding from the *International Statistical Classification of Diseases and Related Health Problesms, Tenth Revision* (*ICD-10*) was used to specify injury subtypes. Injury subtype codes included S00 to S09 (injury to the head), S10 to S19 (injury to the neck), S20 to S29 (injury to the thorax), S30 to S39 (injury to the abdomen, lower back, lumbar spine, pelvis, and external genitals), S40 to S49 (injury to the shoulder and upper arm), S50 to S59 (injury to the elbow and forearm), S60 to S69 (injury to the wrist, hand, and fingers), S70 to S79 (injury to the hip and thigh), S80 to S89 (injury to the knee and lower leg), and S90 to S99 (injury to the ankle and foot). Background traffic accident–related *ICD-10* codes included V00 to V09 (injury as pedestrian), V10 to V19 (injury with bicycle), V20 to V29 (injury with motorcycle), V40 to V49 (injury with automobile), V50 to V59 (injury with van), V60 to V69 (injury with truck), V70 to V79 (injury with bus), V80 to V89 (injury with snowmobile, train, or tram), V90 to V99 (injury related to waterway transport), or unspecified traffic accident–related injury. The types of injuries were obtained from patient medical records.

### Visual Quality for Driving Performance

Visual performance for driving was evaluated with a structured questionnaire (eTable 1 in the Supplement). Visual performance parameters were scored semiquantitatively on a questionnaire that has not been validated. Only patients undergoing bilateral implantation either with non-BLF or BLF IOLs between 2015 and 2016 were selected. The patients were interviewed about avoidance of driving due to visual disturbances, halos, avoidance of evening or nighttime driving, visual disturbances from headlights, difficulties in reading road signs and spotting pedestrians, subjective quality of driving, and glare while driving during daytime or evening and nighttime. Quality of driving was scored from 0 to 3 as follows: 0 indicates poor; 1, moderate; 2, good; and 3, excellent. Glare with driving was scored from 0 to 4 as follows: 0 indicates none; 1, rarely; 2, occasionally; 3, often; and 4, always.

### Cataract Surgery

According to the Finnish National Guidelines for cataract operations, the best-corrected visual acuity for cataract surgery is recommended to be 0.5/0.3 in the better/worse eye, or less by Snellen equivalents, except under some specific circumstances (Current Care Guidelines for Cataracts [https://www.kaypahoito.fi/hoi50035]). The surgical technique used in this study was phacoemulsification (Infinity or Centurion Vision System; Alcon Laboratories, Inc) with a 2.40- to 2.75-mm clear corneal incision.

### Death Reported in the Patient Medical Records and Censoring

When a death certificate is issued, a copy also goes to the population register (Digital and Population Data Services Agency of Finland). As a rule, the date of death is published in a daily update, which is uploaded once a week on Thursdays to the patient medical registry (Lifecare; TietoEVRY). In this update, the data on death cover all patients of a hospital district. When death is reported in the daily data, the Lifecare patient medical record program automatically deletes postmortem appointments and closes referrals. Death was used as a censoring event to improve follow-up precision.

### Follow-up Period

The follow-up started September 3, 2007, and ended on December 14, 2021. The follow-up time is either (1) the difference between the beginning of the follow-up date or the date of the most recent injury, whichever came last, and the date of the first eye surgery or (2) the difference between the date of the second eye surgery and the first injury after the surgery or censoring of the patient, whichever came first. Death and end of follow-up were used as censoring events.

### Statistical Analysis

Unless otherwise specified, data are presented as mean (SD). Only the first event of injury preceding the first eye surgery or after the second eye surgery was recorded, whereas all injury subtypes of the event were included in the injury subtype analysis. Missing data were managed using pairwise omission; that is, for a given analysis, if a patient was missing data on a variable necessary for the analysis, he or she was excluded from that analysis. Other analyses using variables that existed were still possible for that patient. Because very little missing data were encountered during analysis, this method was hardly used. For survival analyses, the follow-up time was counted to the first event or when censoring the data. Patients were censored when death due to causes other than accidents or the end of follow-up was reached. Kaplan-Meier curves were generated, and multivariable Cox proportional hazards regression controlling for age and sex was used to estimate hazard ratios (HRs) for injuries. Statistical analysis was performed using SPSS Statistics, version 27 (IBM Corporation). Two-sided *P* < .05 was considered statistically significant.

## Results

### Injuries Among Patients With Bilateral Non-BLF and BLF IOLs

**Figure 1.**
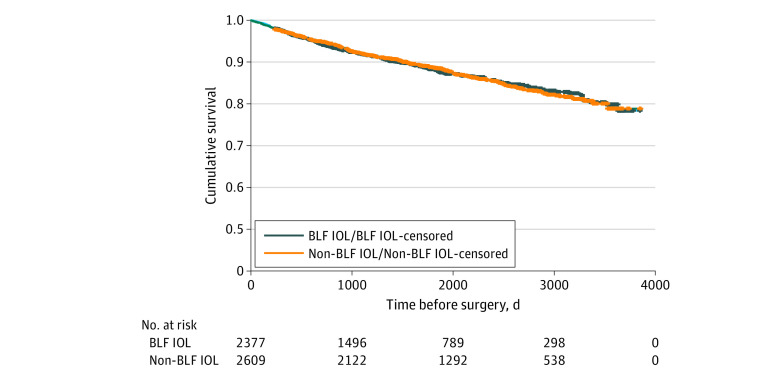
Kaplan-Meier Plot of All-Cause, Injury-Free Survival Preceding Cataract Surgery in the First Eye Data are stratified by blue light–filtering (BLF) and non-BLF intraocular lenses (IOLs). All-cause, injury-free survival was comparable between patients with non-BLF and BLF IOLs (log-rank [Mantel-Cox] *P* = .97).

We included 4986 patients (9972 eyes) who underwent uneventful cataract surgery; 1707 (34.2%) were men and 3279 (65.8%) were women; the mean age was 73.2 (8.6) years at the first surgery and 74.3 (8.8) years at the second. A total of 2609 patients received non-BLF IOLs and 2377 received BLF IOLs (eFigure 1 in the Supplement). The mean follow-up time before the first eye surgery was 1968 (983) days for the non-BLF IOL group and 1618 (1029) days for the BLF IOL group (*P* < .001) (Table 1). Injury-free survival before the first eye surgery was comparable between patients with non-BLF and BLF IOLs (χ^2^_1_ = 0.001; log-rank *P* = .97) (Figure 1). In multivariable Cox proportional hazards regression analysis, age- and sex-adjusted injury-free survival before the first eye surgery remained comparable between patients with non-BLF vs BLF IOLs (HR, 0.95 [95% CI, 0.81-1.13]; *P* = .57). The mean follow-up time after the second eye surgery was 2144 (1042) days for the non-BLF IOL and 2190 (1182) days for the BLF IOL patients (*P* = .15) (Table 1). Baseline variables are presented in Table 1.

**Table 1.** Baseline Characteristics

Characteristic	Treatment group^a^		*P* value^b^
	Non-BLF IOL (n = 2609)	BLF IOL (n = 2377)	
Sex, No. (%)			
Men	978 (37.5)	729 (30.7)	<.001
Women	1631 (62.5)	1648 (69.3)	
Age, mean (SD), y			
At first eye surgery	72.7 (9.3)	76.6 (7.8)	<.001
At second eye surgery	73.7 (9.2)	77.9 (7.8)	<.001
Follow-up, mean (SD), d			
Before first eye surgery	1968 (983)	1618 (1029)	<.001
After second eye surgery	2144 (1042)	2190 (1182)	.15
Ocular comorbidities (in ≥1 eye), No. (%)			
Glaucoma	254 (9.7)	224 (9.4)	.71
PCO^c^	377 (14.4)	358 (15.1)	.54
Retinal detachment	42 (1.6)	21 (0.9)	.02
wAMD	86 (3.3)	102 (4.3)	.07

Abbreviations: BLF, blue light–filtering; IOL, intraocular lens; PCO, posterior capsule opacification; wAMD, wet age-related macular degeneration.

^a^ Patients underwent surgery on both eyes with either hydrophobic monofocal non-BLF IOLs (2609 patients and 5218 eyes) or BLF IOLs (2377 patients and 4754 eyes).

^b^ For 2-group comparisons, qualitative data were analyzed with the 2-factor χ^2^ test and continuous variables with the unpaired *t* test, with *P* < .05 indicating statistical significance.

^c^ Treated with Nd:YAG laser capsulotomy.

Based on *ICD-10* coding, the overall number of injuries after the second eye surgery was 1226; the number of injury subtypes, 1450. Of these, 602 injuries and 727 injury subtypes were among patients with non-BLF IOLs; 624 injuries and 723 injury subtypes were among patients with BLF IOLs (eTable 2 in the Supplement). Injuries were most frequently to the head (n = 312), hip and thigh (n = 289), elbow and forearm (n = 181), and shoulder and upper arm (n = 171) (eTable 2 in the Supplement). None of the ocular comorbidities—namely, glaucoma, posterior capsule opacification, retinal detachment, and wet age-related macular degeneration—had a significant association with the number of injuries (eFigure 2 in the Supplement).

**Figure 2.**
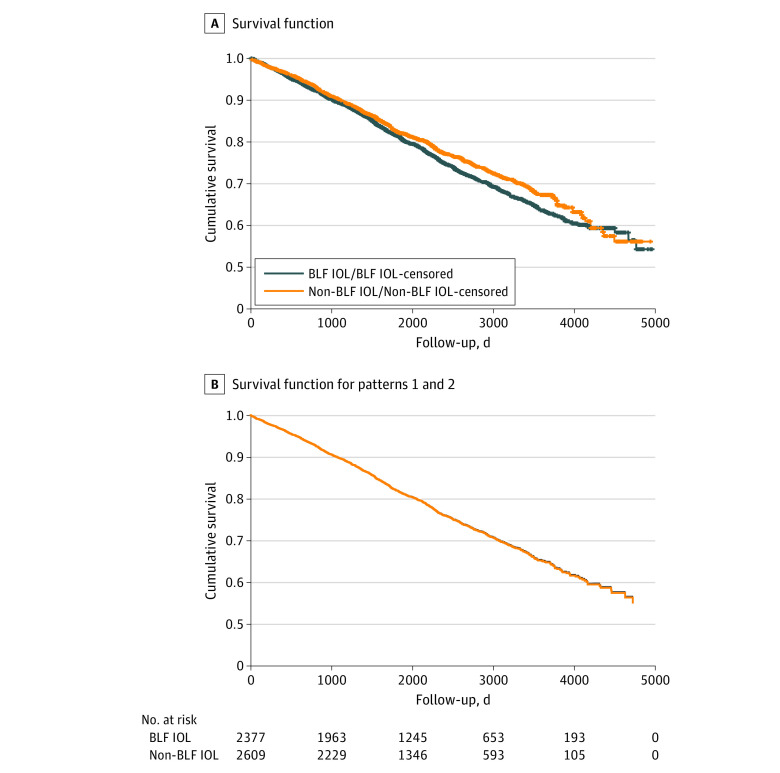
Kaplan-Meier and Multivariable Cox Proportional Hazards Regression Plot of All-Cause, Injury-Free Survival After Cataract Surgery in the Second Eye Data are stratified by blue light–filtering (BLF) and non-BLF intraocular lenses (IOLs). A, All-cause, injury-free survival was comparable between patients with non-BLF and BLF IOLs (log-rank [Mantel-Cox] *P* = .07). B, In multivariable Cox proportional hazards regression analysis controlling for age and sex, the type of IOL (BLF vs non-BLF) was not associated with the all-cause injury rates (hazard ratio, 0.99 [95% CI, 0.88-1.11]; *P* = .85).

On univariate analysis, the incidence of all-cause, injury-free survival after the second eye surgery tended to be lower among patients with BLF IOLs when compared with non-BLF IOLs (log-rank *P* = .07) (Figure 2A). In multivariable Cox proportional hazards regression analysis controlling for age and sex, women tended to have lower incidences of injuries compared with men (HR, 0.89 [95% CI, 0.79-1.01]; *P* = .07), and age at the time of the second eye surgery was associated with a higher rate of injuries (2.6% for every year; HR, 1.03 [95% CI, 1.02-1.03]; *P* < .001), whereas the type of IOL (BLF vs non-BLF IOL) was not associated with the all-cause injury rates (HR, 0.99 [95% CI, 0.88-1.11]; *P* = .85) (Figure 2B). In a more detailed analysis based on *ICD-10* coding, none of the injury subtypes were associated with the IOL’s BLF properties (eTable 3 and eFigure 3 in the Supplement).

**Figure 3.**
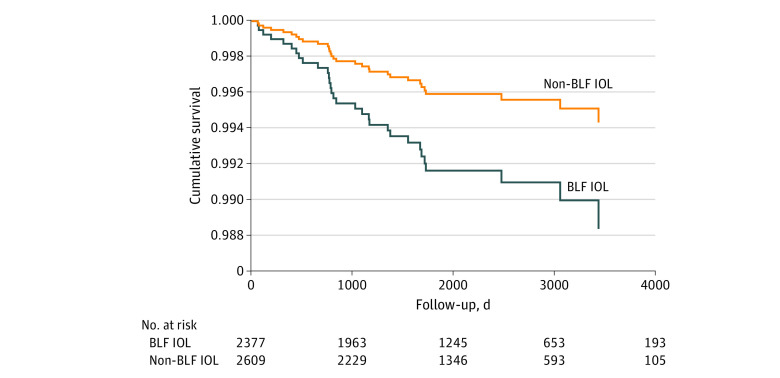
Multivariable Cox Proportional Hazards Regression Plot of Traffic Accident–Related, Injury-Free Survival After Cataract Surgery in the Second Eye Data are stratified by blue light–filtering (BLF) and non-BLF intraocular lenses (IOLs) and controlled for age and sex. In multivariable Cox proportional hazards regression analysis, the type of IOL (BLF vs non-BLF IOLs) was not significantly associated with the traffic accident–related injury rates (hazard ratio, 2.06 [95% CI, 0.97-4.35]; *P* = .06).

Next, we analyzed traffic accident–related injuries (based on *ICD-10* coding) after the second eye surgery between the types of IOL (eTable 2 in the Supplement). In total, we found 31 traffic accident–related injuries, of which 12 were among patients with bilateral non-BLF IOLs and 19 among patients with bilateral BLF IOLs. Traffic accident–related injuries occurred a mean (SD) of 1077 (821) [range, 61-3452] days after the second eye surgery. In univariate analysis, there were no significant differences in traffic accident–related injuries between the patients with BLF IOLs compared with non-BLF IOLs (log-rank *P* = .12). In multivariable Cox proportional hazards regression analysis controlling for age and sex, women had lower incidences of traffic accidents compared with men (HR, 0.41 [95% CI, 0.20-0.84]; *P* = .02), whereas age at the time of the second eye surgery (HR, 0.98 [95% CI, 0.94-1.02]; *P* = .28) and BLF vs non-BLF IOLs (HR, 2.06 [95% CI, 0.97-4.35]; *P* = .06) (Figure 3) were not significantly associated with traffic accident rates.

### Subjective Visual Quality for Driving With Bilateral Non-BLF and BLF IOLs

We included 193 patients who underwent uneventful cataract surgery and were currently driving a car in this analysis. Of these, 102 patients had non-BLF IOLs and 91 patients had BLF IOLs (eFigure 4 in the Supplement). Baseline variables are presented in eTable 4 in the Supplement.

Subjective visual performance parameters for driving were all comparable between the non-BLF and BLF IOL groups (Table 2) except for glare at driving in the dark (evening and night), which was impaired among 9 of 80 patients with BLF IOLs compared with 0 of 83 with non-BLF IOLs (*P* < .001) (Table 2).

**Table 2.** Visual Performance for Driving in Patients Undergoing Implantation With Either Bilateral Non-BLF or BLF IOLs

Variable	No. (%) of patients^a^		*P* value^b^
	Non-BLF IOL (n = 102)	BLF IOL (n = 91)	
Avoid driving owing to visual disturbances	40 (39.2)	33 (36.3)	.63
Avoid driving at evening or night	19 (18.6)	11 (12.1)	.21
Halos	30 (29.4)	18 (19.8)	.12
Visual disturbances from headlights	20 (19.6)	22 (24.2)	.49
Difficulties			
Reading road signs	5 (4.9)	7 (7.7)	.42
Spotting pedestrians	4 (3.9)	4 (4.4)	.87
Quality of driving			
Poor	1 (1.0)	1 (1.1)	>.99
Moderate	32 (31.4)	34 (37.4)	
Good	57 (55.9)	46 (50.5)	
Excellent	12 (11.8)	10 (11.0)	
Glare at driving in daytime			
None	71 (69.6)	66 (72.5)	.57
Rarely	20 (19.6)	9 (10.0)	
Occasionally	10 (9.8)	11 (12.1)	
Often	0	3 (3.3)	
Always	1 (1.0)	2 (2.2)	
Glare at driving at evening or night^c^			
None	55 (66.3)	47 (58.7)	<.001
Rarely	12 (14.5)	9 (11.3)	
Occasionally	10 (12.0)	8 (10.0)	
Often	6 (7.2)	7 (8.7)	
Always	0	9 (11.3)	

Abbreviations: BLF, blue light–filtering; IOL, intraocular lens.

^a^ For 2-group comparisons, binary logistic regression was used for dichotomous outcome measures and ordinal regression for ordinal outcome measures. Only patients undergoing implantation with either non-BLF or BLF IOLs between 2015 and 2016 were selected. Visual performance for driving was evaluated with a structured questionnaire and scored semiquantitatively.

^b^ *P* < .05 indicated statistical significance.

^c^ Includes 163 patients (83 with non-BLF and 80 with BLF IOLs).

## Discussion

In this large group of individuals undergoing cataract surgery, nearly one-half received BLF IOLs, whereas the other half received clear IOLs. The risk of injuries during a long follow-up period after bilateral cataract surgery was assessed using the medical coding system, and individuals undergoing surgery between 2015 and 2016 were also interviewed about difficulties with driving. Within the mean follow-up period of more than 2000 days (>5 years), the cumulative injury-free survival was not different between patients with BLF and non-BLF IOLs. Patient age at the second eye surgery was the most important variable influencing the risk of injury. Furthermore, despite higher levels of glare while driving in the evening or night, the BLF IOLs were not worse than non-BLF IOLs in terms of comfort while driving.

Blue light–filtered IOLs have been widely used in clinical practice for more than 20 years and have been implanted in millions of patients with cataracts worldwide. Still, very little evidence on the association of BLF IOLs with night driving and contrast sensitivity is available.^15,16^ Scotopic contrast sensitivity at low and high spatial frequencies is known to decrease with age,^17^ whereas the rod-mediated dark adaptation progressively becomes slower.^18^ The age-related decline in scotopic sensitivity is most severe for short-wavelength light.^19^ This decrease in scotopic visual function contributes to the increased risk of falling^8^ and difficulties with driving and reading road signs at night among older individuals.^9^ The BLF IOLs filter out 67% to 83% of violet light (400-440 nm) and 27% to 40% of blue light (440-500 nm), depending on their dioptric power,^7^ compared with minimal filtration by standard UV-blocking IOLs. The decrease in spectral transmission is particularly evident in scotopic conditions; Schwiegerling^20^ has shown that BLF IOLs decrease the total transmission by 25.5% when compared with standard UV-blocking IOLs. Thus, there would be grounds for severe concern that IOLs that filter short-wavelength visible light selectively effect the ability to see in the dark. Several studies^10,11^ have evaluated contrast sensitivity in BLF IOLs; however, only 1 study^21^ has assessed the low-illuminance contrast sensitivity. Bandyopadhyay et al^21^ reported nonsignificant differences in photopic (85 cd/m^2^) and mesopic (3 cd/m^2^) contrast sensitivity between clear and yellow- and orange-tinted BLF IOLs. Mainster and Turner^16^ emphasized that BLF IOLs cannot improve contrast sensitivity because contrast transfer at middle to high spatial frequencies depends on wavelengths of 500 to 600 nm, which are unaffected by yellow chromophores.

Data on problems with driving at night can be obtained from vision-related quality-of-life questionnaires. These questionnaires were developed to evaluate a broad range of patient-reported outcomes.^22^ Some of the questionnaires evaluate problems with driving, for example, the National Eye Institute Visual Function Questionnaire 25 or the 14-item visual function index.^23^ Nevertheless, they were not specifically designed to assess difficulties or dysphotopsia during driving. In the study by Espindle et al,^5^ driving scores in the National Eye Institute Visual Function Questionnaire 25 were not different between patients with BLF and non-BLF IOLs. Hammond et al^24^ reported that photostress recovery time and heterochromatic contrast threshold were significantly better in patients with BLF IOLs. Gray et al^25^ found that the safety margin (time to collision minus time taken to turn at an intersection with oncoming traffic) was greater in the BLF group than in the control group. None of these studies analyzed the influence of age as a confounding factor, and both were experimental studies not evaluating real-life outcomes. Of note, the US Food and Drug Administration’s Centers for Medicare & Medicaid Services^26^ concluded that the manufacturer-supported, product-biased studies^24,25^ failed to demonstrate that BLF IOLs produce clinically meaningful improved outcomes in driving safety under glare conditions. Furthermore, a recent study^27^ showed that blue light–blocking spectacles do not improve pedestrian detection or reduce headlight glare in nighttime driving situations. Our study has shown that patients with BLF IOLs experienced more glare at nighttime driving, whereas other subjective disturbances were comparable between the patients with BLF and non-BLF IOLs.

A systematic review and meta-analysis by Desapriya et al^28^ found a nonsignificant reduction in the incidence of falls after expedited cataract surgery. Their review included 2 studies that did not present or analyze the implanted IOL type.^29,30^ In those studies, the risk of injury was ascertained by a diary; in contrast, our investigation was based on medical coding systems. The use of coding indicates that we only examined falls that were serious enough to require hospitalization. Therefore, the incidence of falls and injuries might be underestimated; however, this outcome is unlikely to bias the results. A study by Gadzhanova et al^31^ analyzed the risk of injuries and falling in 2 cohorts depending on the type of IOL—monofocal and multifocal. The risk of injury and falls was nonsignificantly lower in the multifocal IOL cohort compared with the monofocal IOL cohort. Some randomized clinical trials^23,32^ reported on other outcomes after cataract surgery such as visual acuity, complications, or quality of life. Still, the risk of falls and injuries has not been clearly addressed and is not adequately assessed, even in quality-of-life questionnaires. Thus, our study provides evidence of this relevant measure.

### Strengths and Limitations

The strengths of our study are that we had access to the patient medical records for every individual in the study, and our database covers a large population and comprehensively captures all hospitalizations for falls and injuries after the cataract surgery. However, our investigation has limitations. First, allocation to the IOL type was not randomized but was instead based on the operating surgeon’s discretion. To overcome this limitation, multivariable analysis was performed to eliminate the influence of demographic parameters such as age and sex. Second, we did not investigate the reason for giving up driving or objectively evaluate the visual disturbances. The potential risk of car accidents has been estimated to be 3 to 4 times higher at night than in daylight^33^; however, because the study took place in Finland, where light levels in winter months are low, outcomes from other geographical latitudes could be different. In this study, cognitive, psychomotor, and behavioral confounding factors were not measured or evaluated when assessing the risk for injury or driving comfort. Furthermore, our results may not generalize to patient populations in other parts of the world with different traffic levels. In addition, we could not determine whether the patient was the driver or a passenger in the car at the time of the accident. Third, all the patients were treated in a single center with similar equipment and criteria for surgery, but other covariates beyond age and sex influencing the surgeons’ decision on the type of IOL (BFL vs non-BLF) could not be controlled. For instance, the Kaplan-Meier curves and Cox proportional hazards regression survival analyses did not assess whether the participants avoided driving at night or whether they walked independently without assistance, 2 important factors affecting the risk of injury. Moreover, ocular comorbidities were registered at any time point, at least in 1 eye of the patient. The timing and the severity of the ocular comorbidities were beyond the scope of the multivariable analyses. High censorship rates were observed that could influence the accuracy of survival times and reduce power. Finally, the study might inherit bias associated with medical coding (eg, unintentional misspecification, unbundling, and upcoding may lead to coding inaccuracy); still, we intended to receive and operate the highest quality and standardized data.^34^

## Conclusions

Prior studies suggested worse night vision with use of BLF IOLs and the potential for more injuries. In this large cohort study of patients who underwent uneventful cataract surgery in both eyes, despite higher levels of glare at driving in the evening or night, BLF IOLs were not associated with worse outcomes in terms of risk of injury and comfort with driving. These findings could broaden our understanding of the potential advantages and disadvantages of short-wavelength filtration of IOLs.

## Notes

Supplement. **eTable 1.** Structured Questionnaire for Visual Performance for Driving **eTable 2.** Injuries During the Postoperative Follow-up Period (According to *ICD-10* Classification) **eTable 3.** Age- and Sex-Adjusted Hazard Ratios of Injuries Between the IOL Types According to *ICD-10* Classification **eTable 4.** Baseline Variables **eFigure 1.** Study Flowchart **eFigure 2.** Effect of Ocular Comorbidities on Injuries After Cataract Surgery in the Second Eye **eFigure 3.** Multivariable Cox Proportional Hazards Regression Plots of Injury Subtype–Free Survival After Cataract Surgery in the Second Eye According to Type of IOL, Controlling for Age and Sex **eFigure 4.** Study Flowchart for 2015 to 2016
