# Supplementary material for: Association of Blue Light–Filtering Intraocular Lenses With All-Cause and Traffic Accident–Related Injuries Among Patients Undergoing Bilateral Cataract Surgery in Finland
Source: JAMA Netw Open. 2022 Aug 17;5(8):e2227232. doi: 10.1001/jamanetworkopen.2022.27232 (PMC9386539; doi:10.1001/jamanetworkopen.2022.27232)
Supplement: Supplement. — eTable 1. Structured Questionnaire for Visual Performance for Driving eTable 2. Injuries During the Postoperative Follow-up Period (According to ICD-10 Classification) eTable 3. Age- and Sex-Adjusted Hazard Ratios of Injuries Between the IOL Types According to ICD-10 Classification eTable 4. Baseline Variables eFigure 1. Study Flowchart eFigure 2. Effect of Ocular Comorbidities on Injuries After Cataract Surgery in the Second Eye eFigure 3. Multivariable Cox Proportional Hazards Regression Plots of Injury Subtype–Free Survival After Cataract Surgery in the Second Eye According to Type of IOL, Controlling for Age and Sex eFigure 4. Study Flowchart for 2015 to 2016 [file jamanetwopen-e2227232-s001.pdf]

Supplementary Online Content

Kanclerz P, Hecht I, Cunha M, Knyazer B, Laine I, Tuuminen R. Association of blue light–filtering intraocular lenses with all-cause and traffic accident–related injuries among patients undergoing bilateral cataract surgery in Finland. *JAMA Netw Open*. 2022;5(8):e2227232. doi:10.1001/jamanetworkopen.2022.27232

eTable 1. Structured Questionnaire for Visual Performance for Driving
eTable 2. Injuries During the Postoperative Follow-up Period (According to *ICD-10* Classification)
eTable 3. Age- and Sex-Adjusted Hazard Ratios of Injuries Between the IOL Types According to *ICD-10* Classification
eTable 4. Baseline Variables
eFigure 1. Study Flowchart
eFigure 2. Effect of Ocular Comorbidities on Injuries After Cataract Surgery in the Second Eye
eFigure 3. Multivariable Cox Proportional Hazards Regression Plots of Injury Subtype–Free Survival After Cataract Surgery in the Second Eye According to Type of IOL, Controlling for Age and Sex
eFigure 4. Study Flowchart for 2015 to 2016

This supplementary material has been provided by the authors to give readers additional information about their work.

© 2022 Kanclerz P et al. *JAMA Network Open*.

eTable 1. Structured Questionnaire for Visual Performance for Driving*

Items	Response options
Driving experience	number in years
Driving frequency	1=less than once a week<br>2=more than once a week<br>3=daily
Accidents during last 5 years	no / yes
Someone recommended to quit driving	no / yes
Quality of driving	0=poor<br>1=moderate<br>2=good<br>3=excellent
Avoid driving due to visual disturbances	no / yes
Glare at driving: daytime	0=no<br>1=rarely<br>2=occasionally<br>3=often
Avoid driving at evening/night	no / yes
Glare at driving: evening/night	0=no<br>1=rarely<br>2=occasionally<br>3=often
Halos	no / yes
Visual disturbances from headlights	no / yes
Difficulties reading road signs	no / yes
Difficulties spotting pedestrians	no / yes

*The questionnaire has not been validated.

© 2022 Kanclerz P et al. *JAMA Network Open*.

eTable 2. Injuries During the Postoperative Follow-up Period (According to *ICD-10* Classification)

Injuries to the:	Total N=	Non-BLF IOL N=	BLF IOL N=
S00-S09 – head	312	148	164
S10-S19 – neck	15	6	9
S20-S29 – thorax	74	34	40
S30-S39 – abdomen*	79	41	38
S40-S49 – shoulder and upper arm	171	84	87
S50-S59 – elbow and forearm	181	99	82
S60-S69 – wrist, hand, fingers	104	62	42
S70-S79 – hip and thigh	289	131	158
S80-S89 – knee and lower leg	167	88	79
S90-S99 – ankle and foot	58	34	24
V00- – traffic-accident-related	31	12	19

Data are given as absolute numbers. Only the first event of injury after the second eye surgery was recorded. Patients were operated on both eyes with either hydrophobic monofocal non-BLF IOLs (2609 patients; 5218 eyes) or BLF IOLs (2377 patients; 4754 eyes). BLF IOL; blue-light-filtering intraocular lens. S30-S39 Injuries to the abdomen, lower back, lumbar spine, pelvis, and external genitals.

© 2022 Kanclerz P et al. *JAMA Network Open*.

eTable 3. Age- and Sex-Adjusted Hazard Ratios of Injuries Between the IOL Types According to *ICD-10* Classification

Injuries to the:	BLF vs. non-BLF IOL	P-value
S00-S09 – head	1.056 (0.839-1.328)	0.644
S10-S19 – neck	1.851 (0.598-5.723)	0.285
S20-S29 – thorax	1.143 (0.712-1.833)	0.580
S30-S39 – abdomen*	0.816 (0.518-1.285)	0.380
S40-S49 – shoulder and upper arm	0.979 (0.719-1.334)	0.894
S50-S59 – elbow and forearm	0.789 (0.583-1.067)	0.124
S60-S69 – wrist, hand, fingers	0.735 (0.491-1.103)	0.137
S70-S79 – hip and thigh	0.963 (0.759-1.220)	0.752
S80-S89 – knee and lower leg	0.958 (0.699-1.311)	0.787
S90-S99 – ankle and foot	0.730 (0.425-1.254)	0.255
V00- – traffic-accident-related	2.057 (0.973-4.349)	0.059

Data are given as adjusted HR with 95% CI. Patients were operated on both eyes with either hydrophobic monofocal non-BLF IOLs (2609 patients; 5218 eyes) or BLF IOLs (2377 patients; 4754 eyes). BLF IOL; blue-light-filtering intraocular lens. S30-S39 Injuries to the abdomen, lower back, lumbar spine, pelvis, and external genitals.

© 2022 Kanclerz P et al. *JAMA Network Open*.

eTable 4. Baseline Variables

	Non-BLF (N=102)	BLF (N=91)	P-value
Gender (male:female)	63:39	49:42	0.266
Age (years)	74.5±6.7	76.3±5.7	0.048*
Hypertension	42 (41%)	45 (49%)	0.249
Diabetes	29 (28%)	30 (33%)	0.495
Cardiovascular disease	27 (26%)	24 (26%)	0.638
Dry eye disease	33 (32%)	33 (36%)	0.460
Glaucoma	8 (8%)	13 (14%)	0.151
wAMD	1 (1%)	2 (2%)	0.437
Driving experience			
Driving experience (years)	52.8±9.7	54.8±9.7	0.149
Current driving frequency (1 to 3)	4:45:53	9:41:41	0.202
Someone recommended to quit driving	26 (25%)	16 (18%)	0.316
Accidents during last 5 years	5 (5%)	2 (2%)	0.184

Data is given as mean ± SD for continuous variables and absolute numbers with proportions for categorical variables. For two-group comparisons, continuous and normally distributed data was analyzed with the Student's T-test, and non-parametric data with the Mann-Whitney U test. Categorical data was analyzed with the Pearson's chi-square test. Current driving frequency was scored semi-quantitatively from 1 to 3 as follows: 1=less than once a week, 2=more than once a week, 3=daily. BLF; blue-light filtering, IOL; intraocular lens. *P<0.05.

© 2022 Kanclerz P et al. *JAMA Network Open*.

eFigure 1. Study Flowchart

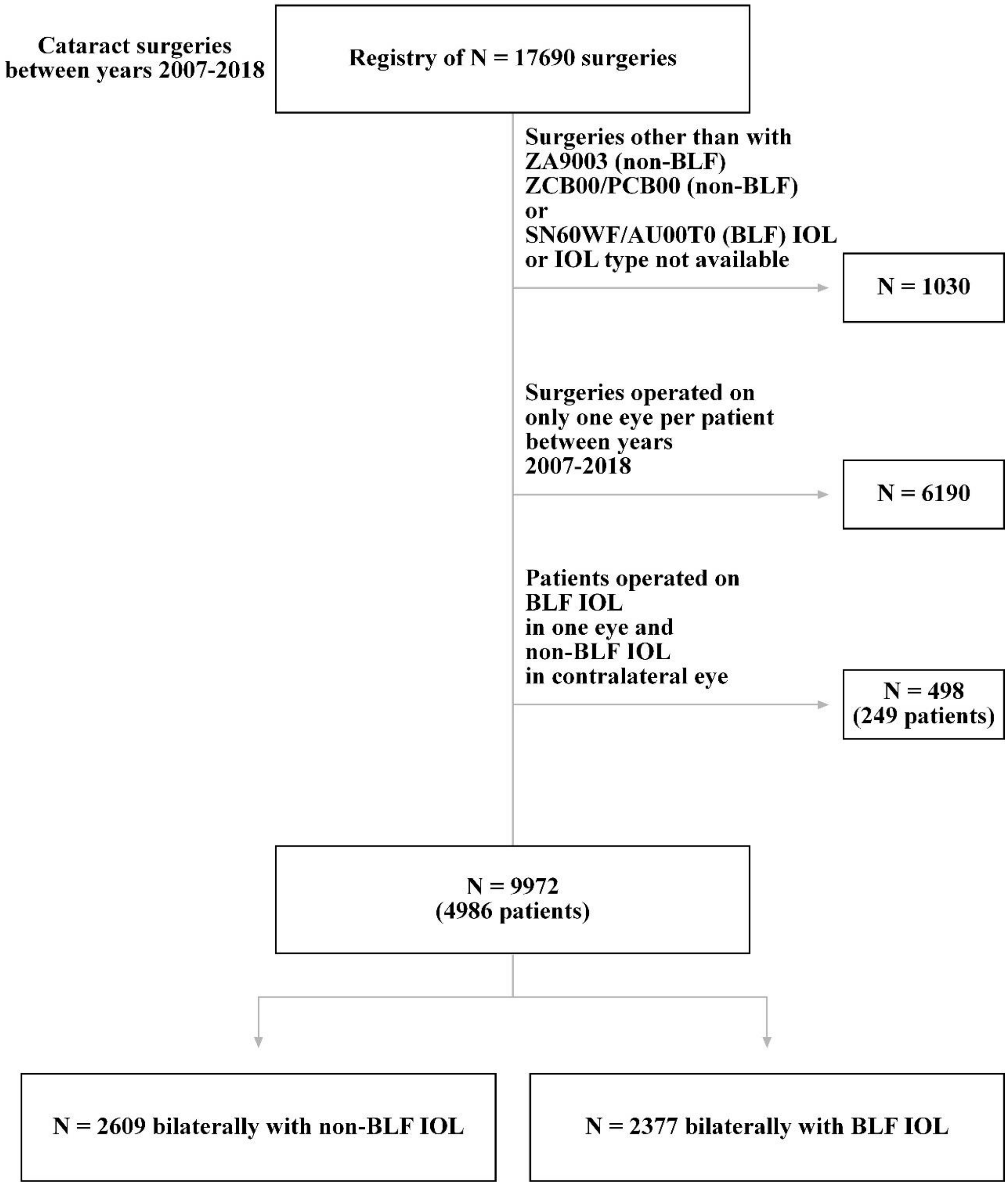

eFigure 1. *Study flow chart.*

Registry of patients operated bilaterally with either non-BLF or BLF IOLs between years 2007 and 2018. BLF; blue-light filtering, IOL; intraocular lens.

© 2022 Kanclerz P et al. *JAMA Network Open*.

eFigure 2. Effect of Ocular Comorbidities on Injuries After Cataract Surgery in the Second Eye

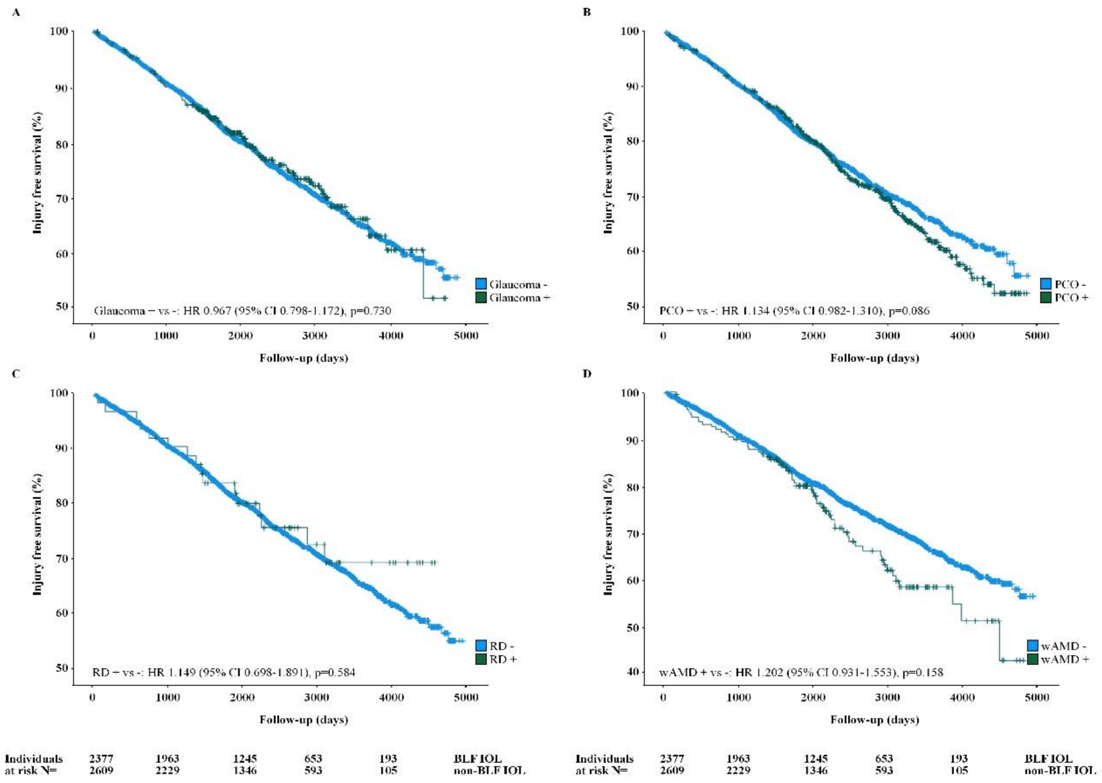

eFigure 2. *Effect of ocular comobidities on injuries after the second eye cataract surgery.*

Univariate Kaplan-Meier plots (log-rank [Mantel-Cox] test) with age- and gender adjusted hazard ratios for injuries after the second eye cataract surgery according to the ocular comorbidities. PCO; posterior capsule opacification, RD; retinal detachment, wAMD; wet age-related macular degeneration.

© 2022 Kanclerz P et al. *JAMA Network Open*.

eFigure 3. Multivariable Cox Proportional Hazards Regression Plots of Injury Subtype–Free Survival After Cataract Surgery in the Second Eye According to Type of IOL, Controlling for Age and Sex

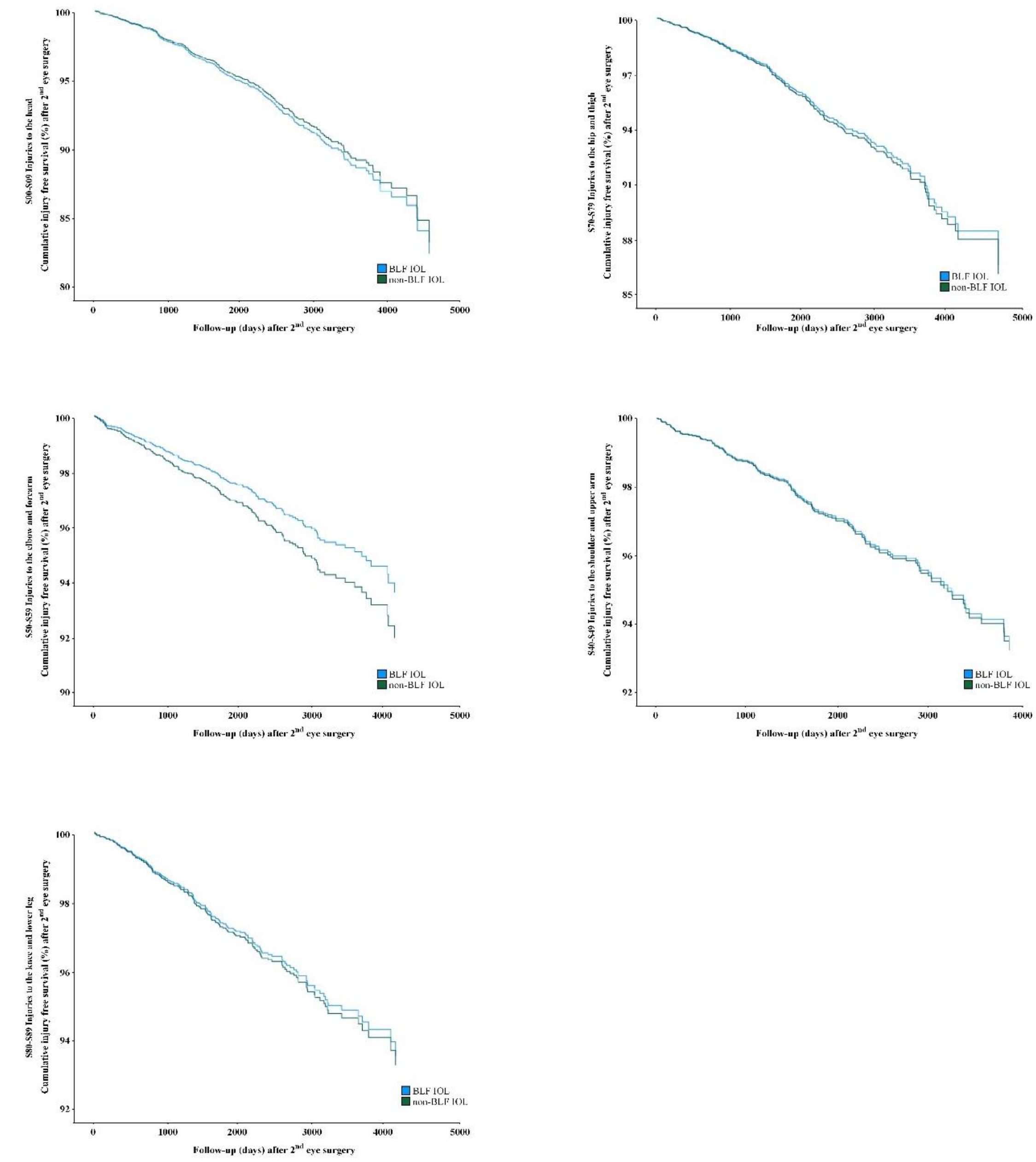

Individuals at risk N=	2377	1963	1245	653	193	BLF IOL
	2609	2229	1346	593	105	non-BLF IOL

eFigure 3. *Multivariate Cox regression plots of injury subtype free survival after the second eye cataract surgery according to the type of IOL, controlling for age and gender.*

Age- and gender adjusted multivariate Cox regression plots of top-five injuries between the IOL types according to ICD-10 classification. BLF; blue-light filtering, IOL; intraocular lens.

© 2022 Kanclerz P et al. *JAMA Network Open*.

eFigure 4. Study Flowchart for 2015 to 2016

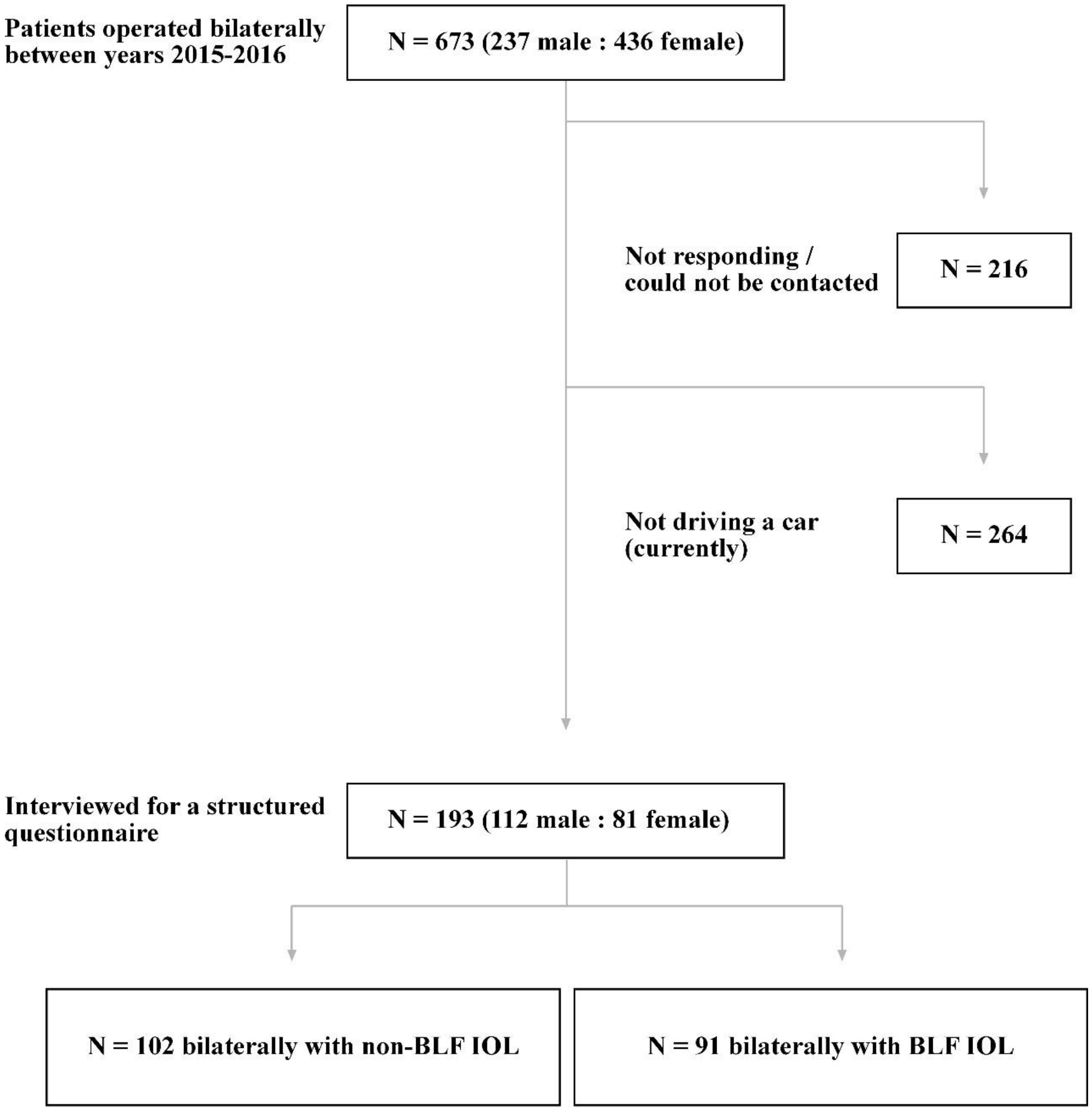

eFigure 4. *Study flow chart.*

Patients interviewed for a structured questionnaire were retrieved from the registry of patients operated bilaterally with either non-BLF or BLF IOLs between years 2015 and 2016. BLF; blue-light filtering, IOL; intraocular lens.

© 2022 Kanclerz P et al. *JAMA Network Open*.
